# Sensitization of capsaicin and icilin responses in oxaliplatin treated adult rat DRG neurons

**DOI:** 10.1186/1744-8069-6-82

**Published:** 2010-11-24

**Authors:** Uma Anand, William R Otto, Praveen Anand

**Affiliations:** 1 Histopathology Unit, Cancer Research UK, London Research Institute, 44 Lincoln's Inn Fields, London WC1A 3PX, U.K; 2 Peripheral Neuropathy Unit, Department of Clinical Neuroscience, Imperial College London, Hammersmith Hospital Campus, Du Cane Road, London W12 0NN, U.K

## Abstract

**Background:**

Oxaliplatin chemotherapy induced neuropathy is a dose related cumulative toxicity that manifests as tingling, numbness, and chronic pain, compromising the quality of life and leading to discontinued chemotherapy. Patients report marked hypersensitivity to cold stimuli at early stages of treatment, when sensory testing reveals cold and heat hyperalgesia. This study examined the morphological and functional effects of oxaliplatin treatment in cultured adult rat DRG neurons.

**Results:**

48 hour exposure to oxaliplatin resulted in dose related reduction in neurite length, density, and number of neurons compared to vehicle treated controls, using Gap43 immunostaining. Neurons treated acutely with 20 μg/ml oxaliplatin showed significantly higher signal intensity for cyclic AMP immunofluorescence (160.5 ± 13 a.u., n = 3, P < 0.05), compared to controls (120.3 ± 4 a.u.). Calcium imaging showed significantly enhanced capsaicin (TRPV1 agonist), responses after acute 20 μg/ml oxaliplatin treatment where the second of paired capsaicin responses increased from 80.7 ± 0.6% without oxaliplatin, to 171.26 ± 29% with oxaliplatin, (n = 6 paired t test, P < 0.05); this was reduced to 81.42 ± 8.1% (P < 0.05), by pretretreatment with the cannabinoid CB2 receptor agonist GW 833972. Chronic oxaliplatin treatment also resulted in dose related increases in capsaicin responses. Similarly, second responses to icilin (TRPA1/TRPM8 agonist), were enhanced after acute (143.85 ± 7%, P = 0.004, unpaired t test, n = 3), and chronic (119.7 ± 11.8%, P < 0.05, n = 3) oxaliplatin treatment, compared to control (85.3 ± 1.7%). Responses to the selective TRPM8 agonist WS-12 were not affected.

**Conclusions:**

Oxaliplatin treatment induces TRP sensitization mediated by increased intracellular cAMP, which may cause neuronal damage. These effects may be mitigated by co-treatment with adenylyl cyclase inhibitors, like CB2 agonists, to alleviate the neurotoxic effects of oxaliplatin.

## Background

Though advances in cancer detection and therapy have significantly advanced life expectancy in cancer patients, quality of life may be severely compromised due to the development of painful neuropathy [1-4]. Chemotherapy-induced peripheral neuropathy is a common, rapidly induced effect observed soon after administration of anti-cancer agents [5-7] resulting in numbness, tingling and pain distributed in a distal stocking-and-glove pattern [8,9]. Oxaliplatin is a highly active antineoplastic agent, licensed for treating colorectal cancer, that contains a platinum complex with a 1,2-diaminocyclohexane (DACH) carrier ligand designed to overcome resistance to other antineoplastic agents [10]. The volume of distribution of platinum is high, due to the lipophilicity of oxaliplatin metabolites, which bind irreversibly to proteins, DNA and other cellular molecules. The terminal half-life of oxaliplatin is long, and neurotoxicity is very common in patients treated with this drug, with 68% experiencing some degree of toxicity. The dose-limiting toxicity is exacerbated by exposure to cold [11-14] at doses of or greater than 135 mg/m^2^, with early development of allodynia and hypersensitivity to heat and cold stimuli [15].

Animal models have reproduced some of the effects of the development of neuropathy after treatment with chemotherapeutic agents, but the mechanism remains unclear. Various treatments have been suggested to provide symptomatic relief for chemotherapy induced neurotoxicity [16,17] but with limited or no efficacy.

Since thermal hypersensitivity is a significant early consequence of oxaliplatin treatment, we investigated the involvement of the ion channels expressed by nociceptors involved in thermosensation. These plasma membrane bound ion channels belong to the transient receptor potential (TRP), superfamily of receptors [18]. TRPV1 (vanilloid subtype 1), is activated by noxious heat (>43°C) [19] capsaicin, low pH, the inflammatory mediators arachidonic acid [20] and bradykinin [21] leading to the perception of pain, and thermal hypersensitivity [22]. The sensitivity and expression of TRPV1 is modulated by the neurotrophins nerve growth factor (NGF) [23-25] and glial cell-line derived neurotrophic factor (GDNF) in rodents [26] and humans [27]. The levels of NGF, GDNF and its receptor ret are increased in injured human peripheral nerves and ganglia [28,29] and in tissues with chronic inflammation [30-32]. TRPV1 expression is upregulated in conditions of chronic pain [33-35] for which it is an important target. As oxaliplatin treated individuals report hypersensitivity to cold stimuli during or soon after infusion, we examined the functional effects of acute oxaliplatin treatment on TRPM8 and TRPA1, two ion channels involved in cool and noxious cold perception respectively. TRPA1 is activated by temperatures less than 17°C, the chemicals mustard oil and cinnamaldehyde (CA), and the cooling agents icilin and menthol; it is expressed in nociceptors, and involved in pain perception although there is inconsistent evidence for its role in cold detection [36-42]. TRPA1 is colocalized with 30% - 50% TRPV1 expressing neurons in rodent [43] and human DRG, where the expression of both channels is increased after injury [44]. Like TRPV1, the responses of TRPA1 to CA demonstrate tachyphylaxis, and are enhanced in the presence of NGF, GDNF (also upregulated in conditions of chronic pain), and NT3 [44]. TRPM8 (subtype melastatin 8), is expressed in a distinct subset of nociceptors, and activated by cool temperature (<25°C), menthol, icilin [45,46], and the carboxyamide derivative WS-12 [47]. Here, we describe the morphological and functional effects of oxaliplatin treatment in cultured adult rat DRG neurons.

## Results

**Figure 1:**
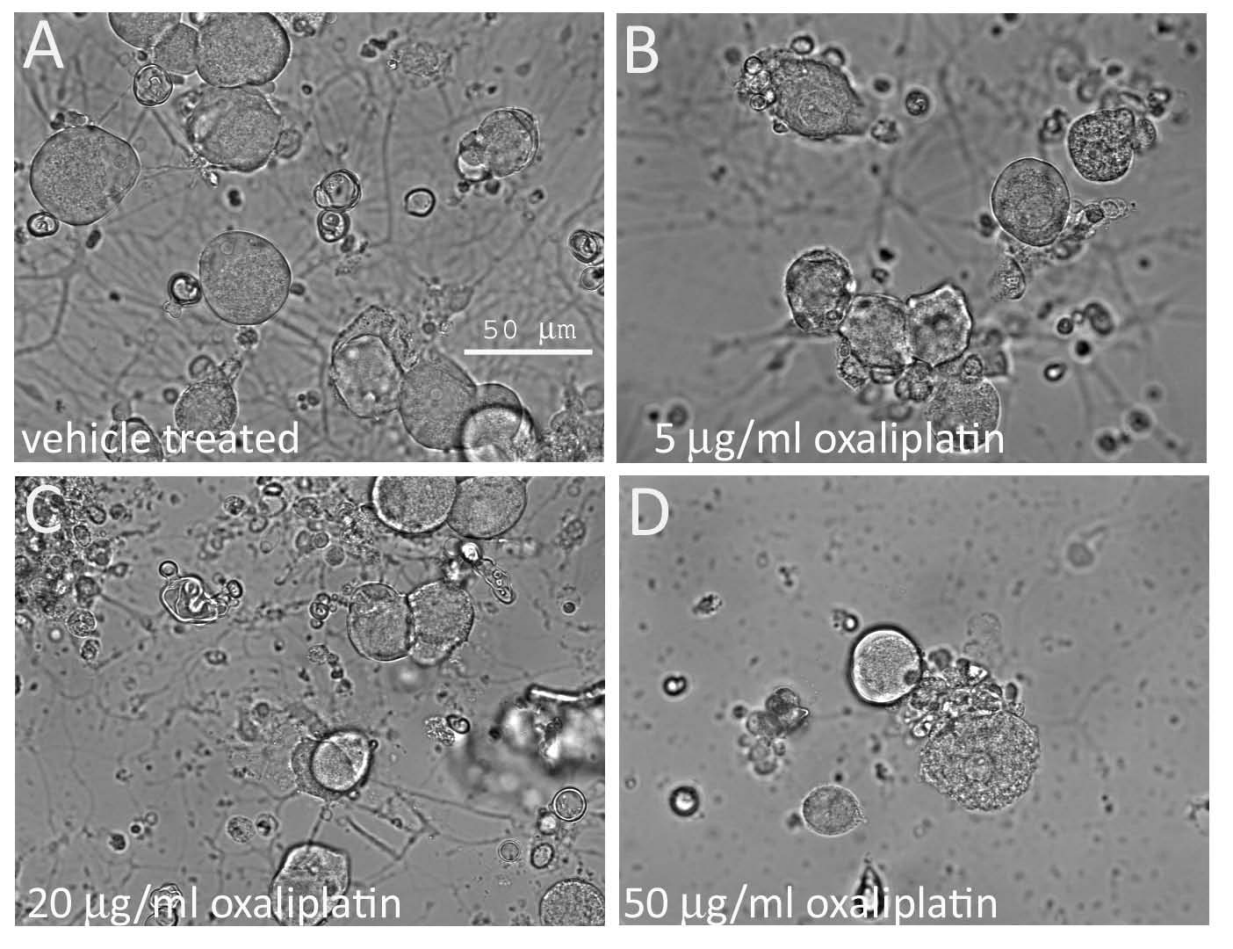
**Phase contrast photomicrographs of cultured adult rat DRG neurons. A**. without oxaliplatin; **B**. after 48 hour treatment with 5 μg/ml oxaliplatin, **C**. 20 μg/ml oxaliplatin and **D**. 50 μg/ml oxaliplatin, showing a dose-related loss of neurite integrity, loss of cell bodies, and accumulation of cell debris. Bar = 50 μm.

**Figure 2:**
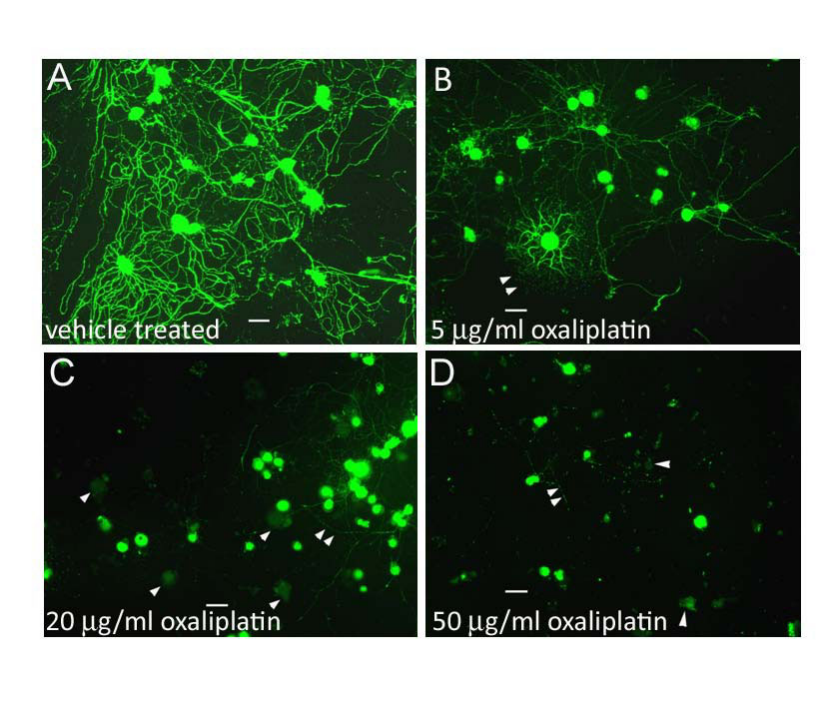
**Gap 43 immunostaining in adult rat DRG neurons *in vitro*. A**. without oxaliplatin treatment, DRG neurons are densely distributed and the cell bodies and neurites are intensely positive for Gap43 immunostaining (green), with long, robust and profusely branched neurites. **B**. Neurons treated with 5 μg/ml oxaliplatin have shorter, less dense neurites (double arrowheads), and reduced intensity of Gap43 immunostaining (double arrowheads). **C**. after 20 μg/ml and **D**. 50 μg/ml oxaliplatin treatment, there is a dose related loss of neurites (double arrowheads), cell bodies (single arrowheads) and loss of Gap43 immunostaining. Bar = 30 μm.

**Figure 3:**
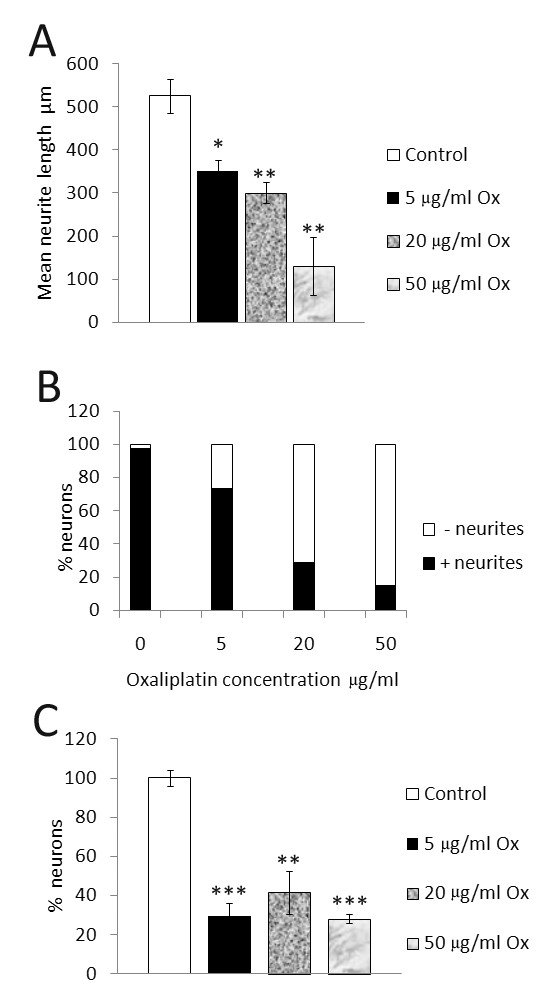
**Morphological effects of oxaliplatin treated neurons**. **A**. Dose-related decrease in maximum neurite length in oxaliplatin treated adult rat DRG neurons *in vitro; *33% reduction after 5 μg/ml oxaliplatin, 42.7% reduction after 20 μg/ml, and 75% reduction after 50 μg/ml oxaliplatin treatment. **B**. Decrease in the percentage of Gap43 positive neurons bearing neurites (solid bars) and increased proportion of neurons without neurites (clear bars) after treatment with increasing oxaliplatin concentration. **C**. The number of neurons surviving after 48 hours oxaliplatin treatment, expressed as a percentage of vehicle treated neurons was significantly reduced after treatment with 5, 20 and 50 μg/ml oxaliplatin.

**Figure 4:**
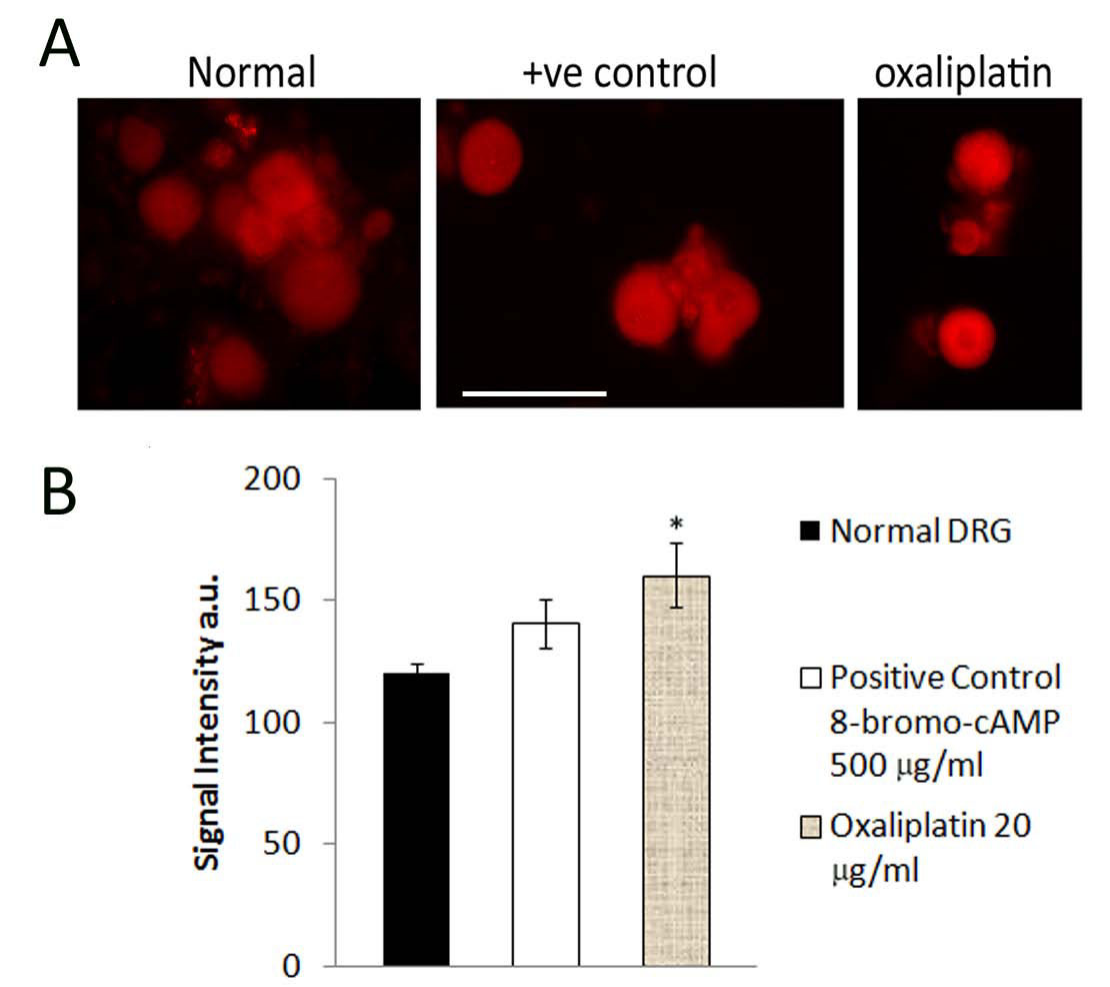
**Increased cAMP signal intensity in oxaliplatin treated neurons. A**. Immunofluorescence images of cAMP positive adult rat DRG neurons acutely treated with 8-bromo-cAMP (positive control), or oxaliplatin for 15 minutes, compared to vehicle treated neurons. Bar = 50 μm. **B**. Fluorescence signal intensity for cAMP immunostaining (arbitrary units), was increased in oxaliplatin treated adult rat DRG neurons compared to positive controls, and significantly higher than vehicle treated neurons.

The cultured neurons had extended neurites by 24 hours after plating, when the oxaliplatin was added, and the morphological effects of oxaliplatin treatment were apparent with phase-contrast microscopy 24 hours later. Measurements taken 48 hours post oxaliplatin treatment showed reduced neurite length, density and number of surviving neurons, effects that were more pronounced with the higher doses of 20 μg/ml and 50 μg/ml than with the lower dose of 5 μg/ml oxaliplatin (Figure 1). These effects were confirmed by Gap43 immunostaining which showed that oxaliplatin-treated neurons had thinner, shorter, vesiculated neurites, which appeared to be disintegrated, leaving a faintly positive halo of neurite fragments around the cell bodies (Figure 2). Gap43 immunostaining appeared patchy and less intense in oxaliplatin-treated neurons compared with control neurons, and was strongest at the cell body and diminished in the neurites (Figure 2). The maximum average neurite length in control neurons was 525.3 ± 29.1 μm (mean ± s.e.m., average of 3 experiments, total 39 neurons). After 48 hours incubation with 5 μg/ml oxaliplatin, the maximum neurite length was significantly reduced to 351.6 ± 23.7 μm (P < 0.05, total 27 neurons), 300.6 ± 23 μm after 20 μg/ml oxaliplatin (P < 0.01, total 20 neurons), and 130.3 ± 67.6 μm after 50 μg/ml oxaliplatin (P < 0.01, total 10 neurons) (Figure 3A). Data are derived from n = 3 culture preparations for each concentration, in each experiment. The number of Gap43-positive neurons was reduced with increasing oxaliplatin concentration, as was the proportion of neurite-bearing neurons (averages from more than 150 neurons for each concentration, n = 3) (Figure 3B). The number of neurons surviving after oxaliplatin treatment was reduced to 29.6 ± 6.4% (total 460 neurons, P = 0.0008), with 5 μg/ml oxaliplatin, 41.7 ± 11% (total 658 neurons, P = 0.01), with 20 μg/ml oxaliplatin, and 28 ± 2.3% (total 435 neurons, P = 0.0002), with 50 μg/ml oxaliplatin, expressed as a percentage ± s.e.m. of vehicle treated neurons (total 1543 neurons) (Figure 3C). cAMP levels in oxaliplatin treated neurons were observed to be significantly higher (160.5 ± 13 a.u., n = 3, 93 neurons P < 0.05), than the vehicle treated neurons (120.3 ± 4 a.u., P < 0.05, n = 3, 50 neurons) and higher than positive controls treated with 500 μg/ml 8-bromo-cAMP (140.45 ± 10, n = 3, 102 neurons) (Figure 4A and 4B).

### Capsaicin responses

**Figure 5:**
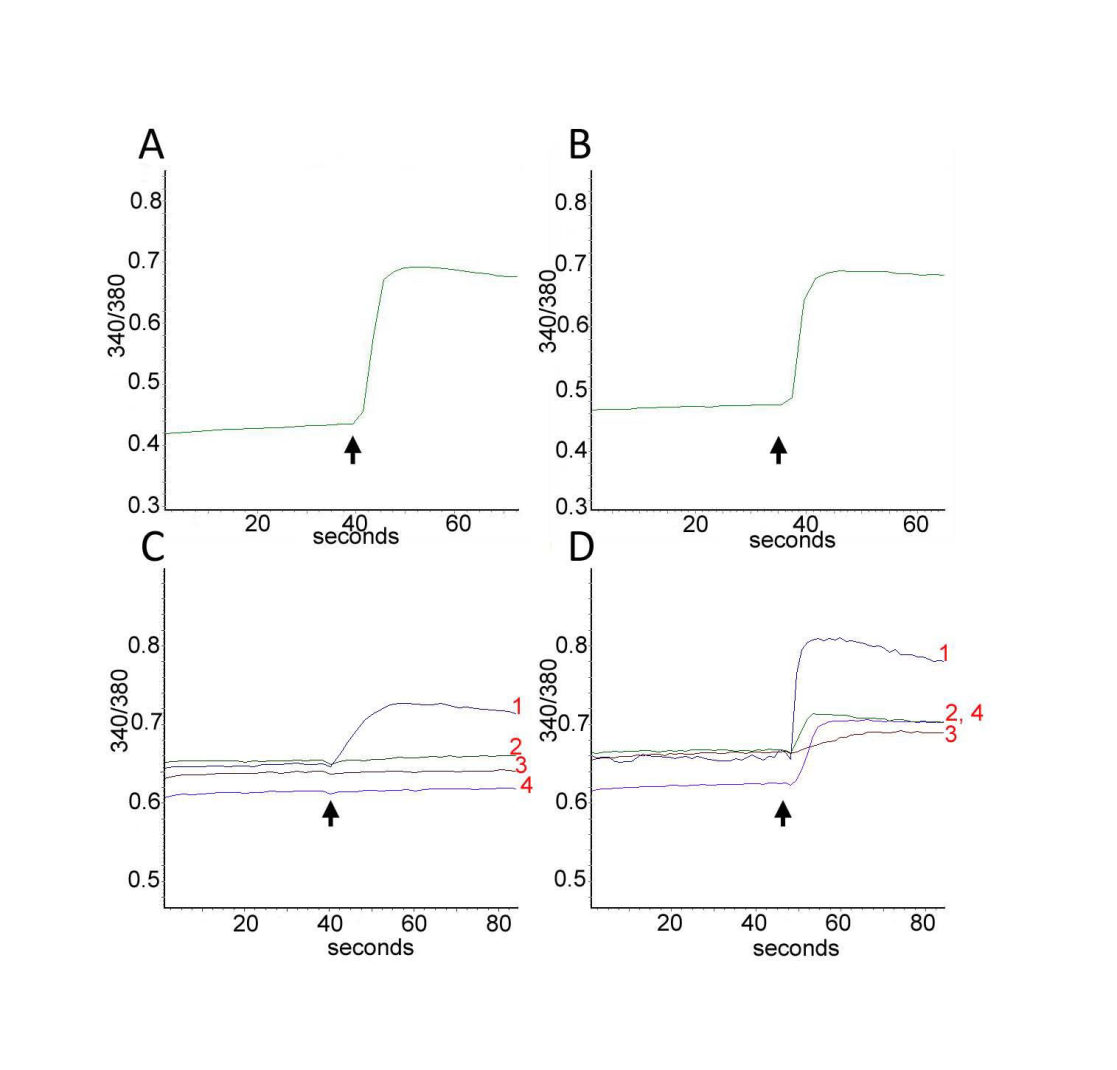
**Sensitization of capsaicin responses by acute oxaliplatin treatment: A. Representative trace showing baseline 340/380 intracellular ratio in a DRG neuron and its increase in response to addition of 200 nM capsaicin (arrow) to test for capsaicin sensitivity**. **B**. Following washout of medium, a second stimulus of 1 μM capsaicin (arrow) after 30 minutes gave a smaller second response compared with the first response (in A), due to desensitization. **C**. Same as in Figure 5A, showing traces 1,2,3,4 from 4 individual neurons, where trace 1 shows increased 340/380 ratio in response to added 200 nM capsaicin (arrow), but not traces 2,3 or 4. **D**. Incubation with 20 μg/ml oxaliplatin for 10 minutes after the first stimulus and washout significantly enhanced the response to 1 μM capsaicin (arrow, trace 1) and evoked responses in previously unresponsive neurons (traces 2 and 4).

**Figure 6:**
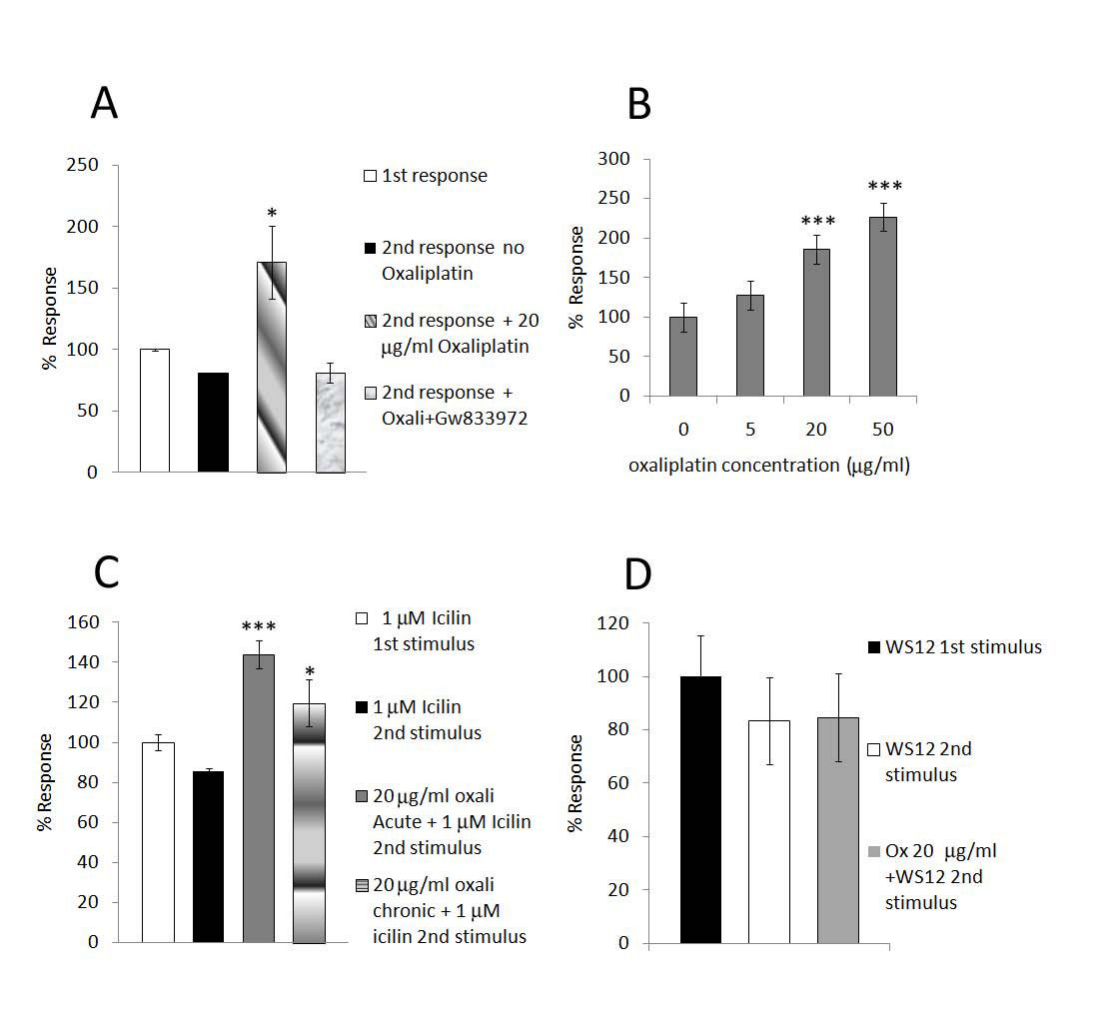
**Sensitization of capsaicin responses after acute (A) and chronic (B) oxaliplatin treatment; Sensitization of responses to icilin (C), but not WS12 (D), after oxaliplatin treatment**. **A**. Graph showing the average magnitude of desensitization (2^nd ^bar) normalised to the first response (1^st ^bar) in vehicle treated neurons. Acute oxaliplatin treatment resulted in significantly enhanced second responses (3^rd ^bar), compared to the first capsaicin response, that was reversed by preincubation with 5 μM CB2 agonist GW833972 (4^th ^bar). **B**. Chronic treatment with oxaliplatin for 48 hours also enhanced responses to 200 nM capsaicin (n.s. at 5 μg/ml oxaliplatin, but highly significant at 20 and 50 μg/ml oxaliplatin). **C**. Vehicle treated neurons responded to 1 μM icilin with calcium influx (1^st ^bar), and a smaller second response following washout (2^nd ^bar). Pretreatment with 20 μg/ml oxaliplatin before the second icilin stimulus significantly enhanced responses to 1 μM icilin (3^rd ^bar), and significantly higher than the second icilin response without oxaliplatin (P = 0.005). Similarly, chronic oxaliplatin treatment (20 μg/ml for 24 hours), also resulted in significantly enhanced second responses to 1 μM icilin compared to those without oxaliplatin (P < 0.05). **D**. Vehicle treated neurons responded to the TRPM8 specific ligand WS-12 (20 μM stimulus), with calcium influx (1^st ^bar). Following washout, a second 20 μM WS-12 stimulus resulted in a reduced response (2^nd ^bar), that was unaffected by the presence of oxaliplatin.

Functional studies using calcium imaging showed that vehicle treated neurons produced a smaller second response to capsaicin (80.7 ± 0.6%, n = 4, 13 neurons, Figures 5B and 6A), compared with the first response (100 ± 0.034%), due to desensitization. Neurons that had been acutely treated with 20 μg/ml oxaliplatin for 10 minutes after the first capsaicin stimulus, showed a significantly enhanced second response to capsaicin (paired t test, P < 0.05, 171.26 ± 29.6%, n = 6, 16 neurons, Figure 5D and 6A), compared to the first response and also when compared with the second response to capsaicin without oxaliplatin (n = 4, 13 neurons, unpaired t Test, P < 0.05). (Figure 5C). Further, some neurons that had not responded to the first response, showed a response to the second capsaicin stimulus after the addition of oxaliplatin (traces 2,3 and 4 in Figure 5D). Preincubation with the CB2 agonist GW833972 eliminated the enhancement seen with oxaliplatin, reducing the second response to 81.42 ± 8.1% of the first response (P < 0.05 n = 3, 12 neurons) (Figure 6A). Neurons treated with 5, 20 or 50 μg/ml oxaliplatin for 48 hours (n = 3), were stimulated with a single application of 200 nM capsaicin, and compared with responses in vehicle treated neurons (n = 5, 27 neurons, Figure 6B). There was a dose-related enhancement of responses, which was significantly greater in neurons treated with 20 (P = 0.003, 22 neurons), and 50 (P = 0.002, 27 neurons) μg/ml oxaliplatin, but not with 5 μg/ml oxaliplatin (n.s. P = 0.1, 22 neurons).

### Icilin responses

In vehicle treated neurons not treated with oxaliplatin, the second response to 1 μM icilin was reduced compared with the first response (85.3 ± 1.7%, n = 3, 8 neurons, Figure 6C), while 5-10 minute treatment with 20 μg/ml oxaliplatin enhanced the second response compared with the first response (143.85 ± 7%, P = 0.004, unpaired t test, n = 3, 11 neurons, Figure 6C). Similarly, chronic treatment (24 hours) with 20 μg/ml oxaliplatin also resulted in significantly enhanced second responses to 1 μM icilin compared to those without oxaliplatin (119.71 ± 11.8%, P < 0.05, n = 3, 11 neurons). Sensitization of icilin responses were unaffected by pretreatment with 5 μM CB2 agonist GW 833972 (not shown).

### WS-12 responses

Responses to the second of a pair of 20 μM WS-12 stimuli, were reduced in vehicle treated neurons compared with the first response (83.39 ± 16.1%, n = 5, 21 neurons, Figure 6D). In the presence of oxaliplatin, there was no change in the average magnitude of the second response (84.6 ± 16.4%, n = 7, 24 neurons), although some neurons responded to the second stimulus but not the first. Spontaneous activity was observed in some neurons after the addition of oxaliplatin. Application of oxaliplatin or vehicle did not activate calcium influx in the neurons, at the concentrations used.

## Discussion

### Morphological effects of oxaliplatin treatment

This study was aimed at understanding the cellular and molecular mechanisms involved in oxaliplatin-induced neuropathy, and showed that adult rat DRG neurons treated with oxaliplatin *in vitro*, undergo dose-dependent loss of neurite length, density, Gap43 expression, and neuronal number (Figures 1, 2, 3). In addition to these morphological changes, increased cAMP levels in oxaliplatin-treated neurons (Figure 4) correlated with functional effects of TRPV1 and TRPA1 sensitization, not observed for TRPM8, after acute and chronic treatment with oxaliplatin (Figure 6). The doses used in our study (5, 20, 50 μg/ml i.e. 12, 48 and 120 μM respectively) were higher than another study (1-10 μg/ml) investigating the effects of oxaliplatin in embryonic (E15) rat DRG [48], but with similar outcomes for neuronal survival and inhibition of neurite outgrowth. Other studies have utilised higher oxaliplatin concentrations of 250 μM on rat DRG neurons [49], and 100 and 500 μM on rat sciatic nerve [50]. Thus, while oxaliplatin targets rapidly dividing tumour cells by the formation of DNA adducts, post mitotic neurons are also affected, possibly by disruption of the synthesis of cytoskeletal elements essential for axon transport of ion channels and other membrane proteins, which are actively recycled from the terminals. Evidence for this may be derived from the diminished Gap43 immunostaining following oxaliplatin treatment. Gap43 is a cytoskeletal, growth associated protein expressed in developing and regenerating DRG neurons [51]; it is rapidly transported to the nerve terminals after synthesis, and is essential for the maintenance of neurites [52]. Oxaliplatin thus appears to have similar morphological effects to cisplatin, taxol and paclitaxel [53-55]. The dose dependent reduction of neurite length indicates damage that could result in disruption of nerve terminals and signal transduction components, and provide a basis for the stocking and glove distribution of sensory abnormalities experienced by chemotherapy recipients. Damaged afferent neurites are capable of sprouting from their peripheral terminals and reconnecting with their peripheral target tissue, so that resolution of dysaesthesia and paraesthesia would be expected to occur with time after discontinuation of chemotherapy. However loss of sensory neurons due to cell death is likely to produce permanent sensory deficits due to the inability of sensory neurons to be replaced, thus creating an imbalance between the surviving fibre types with respect to modality. Further, as injured afferent fibres are capable of generating ectopic signals from their damaged terminals and cell bodies within the DRG [56] chemotherapy induced neuronal damage may lead to chronic pain.

### Oxaliplatin pharmacokinetics

Studies in patients have indicated that oxaliplatin binds irreversibly to plasma proteins and has a long half life which could lead to the cumulative dose related toxicity. Thus while the doses used in our study were higher than the maximum concentration levels (4.81 ± 1.83 μg/ml), which is closest to our lowest dose of 5 μg/ml) observed in plasma ultrafiltrate of patients treated with a single oxaliplatin infusion [57], they may represent the levels present in tissues including peripheral nerves, accumulated during the course of treatment. AUC_0-inf _(area under the curve) values of 278 ± 81 μg/ml*h following a single 4 hour infusion at 130 mg/m^2 ^(cycle 1) have been reported for plasma platinum [57].

### Animal models of chemotherapy mediated neuropathy

Rodents treated with paclitaxel and vincristine developed a partial degeneration of terminal arbours in the epidermis, increased spontaneous discharge of peripheral nerve fibres [58], reduced nerve conduction velocity after cisplatin and paclitaxel administration [59], and thermal hyperalgesia, with upregulation of TRPV1, TRPM8 and TRPA1 mRNA several hours after cisplatin and oxaliplatin treatment [60].

### Functional effects of oxaliplatin treatment on TRPV1, TRPA1/M8

We have studied the functional effects of oxaliplatin on the neuronal characteristic of desensitization (tachyphylaxis) due to repeat stimulation [61-63]. The desensitization observed in our experiments was between the first capsaicin stimulus of 200 nM and the second stimulus of 1 μM, 30 minutes after washout of medium; this protocol has been previously described [64] and the modest desensitization is attributed to the combination of growth factors used for culturing the neurons, and the long gap between the first and second stimuli. However, we observed acute sensitization of TRPV1 within 10 minutes of applying 20 μg/ml oxaliplatin, seen as significantly enhanced capsaicin responses with calcium imaging, compared to the desensitization observed in vehicle treated neurons (Figures 5 and 6A) indicating a modulatory effect of oxaliplatin on the sensitivity of TRPV1. Chronic oxaliplatin treatment (48 hours) also resulted in significantly enhanced dose related responses to 200 nM capsaicin, in contrast to vehicle treated neurons (Figure 6C). Some neurons developed spontaneous activity after oxaliplatin application, or showed a response to the second capsaicin stimulus following oxaliplation addition, but not the first capsaicin stimulus (traces 2, 4, in Figure 5D). Thus oxaliplatin differs from Ara C (cytosine arabinoside - used for treating leukemia), which had the effect of abolishing capsaicin responses by blocking the insertion of TRPV1 ion channels in the cell membrane of human DRG neurons, but causing hypersensitivity when Ara C treatment was stopped, due to the reappearance of TRPV1 in the cell membrane [65]. As oxaliplatin application itself did not result in calcium influx, it appears not to directly activate the TRPV1 receptor, but may bring about the observed effects via a second messenger, such as cAMP, which we found was significantly increased in oxaliplatin treated neurons compared to controls (Figure 4). The rapid sensitization of TRPV1, within minutes of applying oxaliplatin as observed in this study, probably occurs via post translational modification, in a manner similar to that observed in the rapid membrane insertion of TRPV1 ion channels following activation of protein kinase A and phosphorylation of TRPV1 [25,66,67].

### Mechanism of TRPV1 sensitization and effect of CB2 agonist

Since adenylyl cyclase activation is involved in TRPV1 sensitization via cAMP [68] by phosphorylation of TRPV1 [69], we compared cAMP expression in vehicle treated neurons, positive controls i.e. neurons treated with membrane permeant 8-bromo-cAMP, and in neurons acutely treated with oxaliplatin, using immunofluorescence. The significant increase in cAMP observed in oxaliplatin treated neurons, correlated well with the functional data, which showed acute sensitization following oxaliplatin treatment. This observation is strengthened by the abolition of oxaliplatin mediated sensitization of capsaicin responses by preincubation with the CB2 (cannabinoid receptor subtype 2) agonist GW833972 (Figure 6A). CB2 mediated inhibition of capsaicin responses has been shown in rats [70], and guinea pigs [71,72], mediated by TRPV1 dephosphorylation [73]. CB2 receptors are G protein coupled receptors expressed by DRG neurons, and CB2 agonists have been shown to inhibit adenylyl cyclase, and reduce cAMP, to inhibit TRPV1 sensitization in human sensory neurons *in vitro *[64]. On the basis of these results, it can be hypothesized that inhibitors of adenylyl cyclase may be expected to ameliorate TRPV1 mediated hypersensitivity following oxaliplatin chemotherapy. The acute effects of oxaliplatin observed in this study may have been brought about by activation of adenylyl cyclase and a consequent increase in cAMP leading to TRPV1 phosphorylation and sensitization. This is likely to cause further excitotoxicity by increased calcium influx and may be responsible for structural damage such as neurite loss, which could potentially lead to neuropathic pain.

### Effect of oxaliplatin on TRPA1/M8 function

As the infusion of oxaliplatin is accompanied by the symptom of hypersensitivity to cold stimuli in patients, we examined the involvement of the cool receptor TRPM8, and the noxious cold receptor TRPA1, which are activated by the chemicals menthol and icilin [74,75]. Identification of icilin sensitive neurones and examination of their subsequent responses in the presence of oxaliplatin required repeated stimulation with icilin, which resulted in 14.7% reduction of the second response. TRPM8 is subject to desensitization on repeat stimulation [40,76], as is TRPA1. Acute and chronic oxaliplatin treatment resulted in sensitization of icilin responses indicating that either one or both channels could be affected (Figure 6C). In order to identify which of these channels was involved, we used the TRPM8 specific carboxyamide derivative WS-12 [40], but no consistent sensitization of WS-12 responses was observed after oxaliplatin treatment (Figure 6D), indicating that TRPM8 may not be involved in oxaliplatin mediated effects. Our conclusion that oxaliplatin affects TRPA1 rather than TRPM8, is based on the sensitization of icilin responses observed after oxaliplatin treatment but not of the responses using the TRPM8 specific ligand WS12; this is an indirect approach to distinguish between the two ion channels, and may represent a limitation of this study. As the low dose used (1 μM) is closer to the EC_50 _for TRPM8 [40], than for TRPA1, it is possible that TRPM8 rather than TRPA1 is involved in oxaliplatin mediated cold hypersensitivity; this would have to be confirmed by future studies using thermal stimuli.

A clinical effect on nerve fibres expressing TRPA1 could account for early symptomatic cold hypersensitivity, while the cold and heat hyperalgesia on sensory testing may result from sensitization of TRPA1 and TRPV1 ion channels respectively. As the clinical symptoms can be acute, a postranslational mechanism of activation is more likely than changes in gene expression, in the immediate phase.

## Conclusions

This is the first study to describe sensitization of TRPV1 and TRPA1 and/or TRPM8 in a model of oxaliplatin induced neuropathy, the role of cAMP in mediating the sensitization, and the effectiveness of a CB2 agonist in mitigating it.

## Methods

Bilateral DRG from all spinal levels were aseptically harvested from 19 freshly sacrificed adult female Wistar rats (250 grams), in Ham's F12 medium, enzyme digested in 0.2% collagenase (Worthington type IV)/0.5% dispase for 3 hours at 37°C, and dissociated in modified BSF2 medium [77], containing 2% fetal calf serum and soy-bean trypsin inhibitor (1 mg/ml), to obtain a single cell suspension. 100 μl neuronal suspension was plated on collagen (type I, 50 μg/ml), and laminin (20 μg/ml), coated MatTek dishes (glass bottomed plastic petri dishes), in BSF2 medium and incubated at 37°C in a humid environment, for 30 minutes before adding 2 mls BSF2 containing 50 ng/ml each NT3 and GDNF, 100 ng/ml NGF, to each dish. 24 hours later, oxaliplatin, was added to some of the cultures at 5 μg/ml (12 μM), 20 μg/ml (48 μM), 50 μg/ml (120 μM), or vehicle (0.4% distilled water).

### Immunostaining

Cultures were fixed 48 hours after oxaliplatin treatment, with 4% paraformaldehyde (PFA), for 15 minutes, washed in PBS, permeabilised with methanol (-20°C, 3 minutes), washed in PBS and incubated with primary antibody, mouse monoclonal anti Gap43 (1: 200). This was followed by 3 PBS washes and secondary antibody Alexa Fluor 488 (Molecular probes, 1: 200), for 45 minutes at room temperature, washed with PBS, and mounted in Citifluor containing Dabco (antifade agent). Cyclic AMP (cAMP) immuostaining was performed in vehicle treated neurones, and neurons treated with 20 μg/ml oxaliplatin or 500 μM 8-bromo cAMP (Calbiochem, positive controls) for 15 minutes. Following PFA fixation, immunostaining with primary rabbit polyclonal antibody to cAMP (AB306, Millipore, USA, 1:1000), and Alexa 546 secondary antibody, was performed as above. Images were acquired with Smartcapture 3 (Digital Scientific, Cambridge UK), connected to an upright Olympus BX61 microscope, after confirming the absence of immunostaining in negative controls where the primary antibody had been omitted. Adobe Photoshop C53 software was used to measure the maximum neurite lengths in Gap43 immunostained neurons, and cells with and without neurites were counted in each group (n = 3 for each group). Images of cAMP immunostaining were captured using 0.2 seconds exposure time, and fluorescence intensity was measured (arbitrary units a.u.) with Metamorph software (Molecular Devices), imported and analysed with Excel software.

### Calcium imaging - capsaicin responses

Neuronal cultures were loaded with the calcium indicator dye Fura 2 AM, (Molecular Probes, 2 μM, 45 minutes at 37°C), in phenol-red free Hank's balanced salt solution (HBSS), containing 0.1% BSA, washed and incubated in HBSS containing 0.5% BSA for 20 minutes, and baseline changes in bound/unbound calcium ratio (340/380 λem nm), in response to capsaicin stimulation were monitored as previously described [63]. Acute effects of oxaliplatin were determined in normal neurons stimulated with a test dose of 200 nM capsaicin to identify capsaicin sensitivity, followed by washout and a second capsaicin stimulus of 1 μM after 30 minutes; this was compared with the responses to 1 μM capsaicin in a separate group of neurons, preincubated with 20 μg/ml oxaliplatin, or the cannabinoid receptor subtype 2 (CB2), agonist GW 833972 and oxaliplatin, for 10 minutes following the 200 nM test dose of capsaicin and washout. Chronic effects of oxaliplatin were determined by measuring the first response to 200 nM capsaicin in neurons pretreated with oxaliplatin for 24-48 hours and compared with vehicle treated controls.

### Icilin responses

Fura 2 loaded normal untreated neurons were stimulated with a pair of stimuli of 1 μM icilin, separated by washout of medium and a 10 minute rest period. The responses were compared with those obtained after acute application of 20 μg/ml oxaliplatin before the second icilin stimulus. Chronic effects of oxaliplatin treatment were determined in neurons treated with 20 μg/ml oxaliplatin for 24-48 hours, with repeat stimulation using 1 μM icilin.

### WS-12 responses

The TRPM8 specific ligand WS-12 was used in an identical protocol as icilin, with paired stimuli of 20 μM, separated by washout of medium and a 10 minute rest period between the two stimuli. The responses were compared with those from neurons acutely treated with 20 μg/ml oxaliplatin before the second WS-12 stimulus. Students t-test was used to compare groups and results are given as Mean ± SEM, n = number of culture preparations and n.s. indicates 'not significant'. P < 0.05 was set as the level for statistical significance. Capsaicin was freshly prepared in ethanol at 400× final concentration from 20 mM stock solution (in DMSO aliquoted and stored at -20°C). Oxaliplatin was dissolved in sterile distilled water at 5 mg/ml concentration, and stored as aliquots at -20°C, which were freshly thawed prior to use. Icilin and WS-12 stocks were prepared at 400× final concentration in ethanol; all chemicals were purchased from Sigma, U.K. unless stated otherwise. GW833972 is 2-[(2,4-dichlorophenyl)amino]-N-(4- pyridinylmethyl)-4-(trifluoromethyl)-5 pyrimidine carboxamide, CB2 EC50 = 60 nM [kind gift of GlaxoSmithKline, UK]. WS-12 is 2 S,5R-2-Isopropyl-N-(4-methoxyphenyl)-5-methylcyclohexanecarboxyamide. Rats were housed and tissues were harvested according to UK Home Office guidelines.

## Competing interests

The authors declare that they have no competing interests.

## Authors' contributions

UA conceived and designed the study, conducted the studies, analysis and wrote the manuscript. WRO and PA participated in the study design, interpretation of the results and reviewing of the final results. All authors have read and approved the final manuscript.
